# Physical fitness in older women with osteoporosis and vertebral fracture after a resistance and balance exercise programme: 3-month post-intervention follow-up of a randomised controlled trial

**DOI:** 10.1186/s12891-020-03495-9

**Published:** 2020-07-18

**Authors:** Brita Stanghelle, Hege Bentzen, Lora Giangregorio, Are Hugo Pripp, Dawn A. Skelton, Astrid Bergland

**Affiliations:** 1 Institute of Physiotherapy, Faculty of Health Sciences, Oslo Metropolitan University, Oslo, Norway; 2 Department of Kinesiology, University of Waterloo and Schlegel-UW Research Institute for Aging, Waterloo, Canada; 3 School of Health and Life Sciences, Institute of Applied Health Research, Centre for Living, Glasgow Caledonian University, Glasgow, UK

**Keywords:** Osteoporosis, Vertebral fracture, Exercise, Physical fitness, Short-term effects

## Abstract

**Background:**

Exercise is recommended for individuals with vertebral fractures, but few studies have investigated the effect of exercise on outcomes of importance for this population. Post-intervention effects of exercise are even less studied. The objective of this study was to evaluate habitual walking speed and other health-related outcomes after cessation of a 3-month exercise intervention.

**Methods:**

This follow-up study was conducted 3 months post-intervention of a randomised controlled trial. A total of 149 community-dwelling Norwegian women aged 65 years or older, diagnosed with osteoporosis and vertebral fracture were randomised into either exercise or control group. Primary outcome was habitual walking speed at 3 months. Secondary outcomes were other measures of physical fitness – including the Four Square Step Test (FSST), functional reach, grip strength and Senior Fitness Test – measures of health-related quality of life and fear of falling. Herein we report secondary data analysis of all outcomes at 6 months (3 months post-intervention). Data were analysed according to the intention-to-treat principle, linear mixed regression models were employed.

**Results:**

For the primary outcome, habitual walking speed, there was no statistically significant difference between groups (0.03 m/s, 95%CI − 0.02 to 0.08, *p* = 0.271) at the 3-month post-intervention follow-up. For secondary outcomes of physical fitness, statistically significant differences in favour of the intervention group were found for balance using the FSST (− 0.68 s, 95%CI − 1.24 to − 0.11, *p* = 0.019), arm curl (1.3, 95%CI 0.25 to 2.29, *p* = 0.015), leg strength using the 30-s sit to stand (1.56, 95%CI 0.68 to 2.44, *p* = 0.001) and mobility using the 2.45-m up and go (− 0.38 s, 95%CI − 0.74 to − 0.02, *p* = 0.039). There was a statistically significant difference between the groups regarding fear of falling in favour of the intervention group (− 1.7, 95%CI − 2.97 to − 0.38, *p* = 0.011). No differences between groups were observed for health-related quality of life.

**Conclusion:**

The results show the improved effects of a multicomponent exercise programme on outcomes like muscle strength, balance and mobility as well as fear of falling in a group of older women with osteoporosis and vertebral fracture 3 months post-intervention.

**Trial registration:**

ClinicalTrials.gov Identifier: NCT02781974. Registered 25.05.16. Retrospectively registered.

## Background

Vertebral fractures are among the most common fragility fractures caused by osteoporosis [1, 2]. In the European Union in 2010, there were 3.5 million new fragility fractures, and 520,000 of them were vertebral fractures [2]. These numbers are likely an underestimate, as only about two-thirds of vertebral fractures come to clinical attention [3]. The risk of new vertebral fractures increases with the number and severity of prevalent vertebral fractures [3]. Vertebral fractures are associated with increased mortality and morbidity [3, 4] and contribute to back pain, impaired physical function and lower health-related quality of life (HRQoL) [5–8].

Clinical practice guidelines for the management of osteoporosis emphasise the importance of exercise [9, 10]. There is evidence that exercise prevents falls in older people [11]. Exercise can improve physical function, activities of daily living and HRQoL in older people who are frail [12]. However, individuals with vertebral fractures require exercise that is tailored to address safety and related impairments [13]. A recently updated systematic review on the effect of exercise for people with vertebral fracture [14] concluded that there is moderate-quality evidence that exercise improves physical performance. However, few studies have evaluated whether the effects of exercise are sustained after the intervention has ceased [15–18]. The results from a limited number of studies examining follow-up after cessation of exercise in older women with osteoporosis and vertebral fracture were promising. In these individual studies (ranging from 12 weeks to 12 months of follow-up), the sustained effects of exercise were reported on QoL [16]; maximum walking speed, mobility and HRQoL [15]; functional leg muscle strength [18]; and fear of falling [17].

Understanding whether the effects of an exercise intervention persist after cessation is important for several reasons. Sustaining adherence to exercise and thus any benefits from supervised resistance training is difficult without the support and help of staff [19]. The natural decline in physical fitness caused by aging may counteract the gains from an exercise intervention if it is not maintained. Muscle strength gained after an intervention is often lost or decreased in older people after a period of detraining [20]. Similarly, improvements in balance in older adults may also be lost after detraining [21–23]. Conversely, it is possible that participating in an exercise intervention may motivate some participants to maintain exercise outside of the trial to preserve the benefits or see continued improvements.

We have previously reported on a randomised controlled trial [24] of a multicomponent exercise programme informed by exercise recommendations for people with osteoporotic vertebral fracture [13]. In that study, exercise improved muscle strength, balance and fear of falling in older women with osteoporosis and vertebral fracture. However, there was no effect immediately post-intervention on habitual walking speed and HRQoL. It is important to evaluate whether effects of exercise on muscle strength, balance and fear of falling would be sustained upon cessation of the intervention and whether there would be changes in habitual walking speed or QoL at follow-up. Thus, the aim of the current study was to examine the changes to habitual walking speed and other health-related outcomes after cessation of a 3-month exercise intervention compared to a control group.

## Methods

We conducted a single-blinded randomised controlled trial with two arms, with participants allocated to the control or intervention group in a 1:1 ratio. A computer-generated permuted block randomisation scheme provided by a statistician was used to allocate the participants. The scheme was kept and administered by a person not involved with the study participants and was unavailable to others. The block sizes varied from four to eight.

The intervention group attended a resistance and balance exercise programme twice weekly for 12 weeks. The control group was asked to live life as usual [24, 25]. In the period from the end of intervention to the 3-month follow-up, all participants were instructed to live life as usual. That meant continuing with their usual activities and maintaining their usual level of physical activity. The present study reports on follow-up measurements performed 3 months after completing the intervention or control activities (6 months post-randomisation). Detailed information on the study protocol is reported elsewhere [25]. Reporting follows the CONSORT 2010 statement [26], and a CONSORT checklist is provided in the Supplementary Files. The study was approved by the Regional Committee for Medical Research Ethics in South East Norway (Ref. 2014/2050) and is registered with Clinical Trials (NCT02781974).

### Settings and participants

The study was conducted both at facilities at Oslo Metropolitan University in Oslo, Norway and at a physiotherapy clinic near Oslo. Recruitment of participants was done from outpatient clinics for osteoporosis at two different public hospitals and a private speciality outpatient clinic in or nearby Oslo. The recruitment period was from January 2016 to April 2018, with the last follow-up test completed in October 2018. The recruitment ended once the desired number of participants was reached. All patients provided written informed consent prior to baseline testing.

The inclusion criteria were female sex, age 65 years or older, community dwelling and a diagnosis of osteoporosis defined as a T score ≤ − 2.5 SD at the femoral neck or lumbar spine verified by dual X-ray absorptiometry (DXA). To be included, the participants had to have at least one prevalent vertebral fracture classified as grade 1, 2 or 3 by the Genant method [3] or verified by DXA-based vertebral fracture assessment or via X-ray by medical doctors not involved in the study. Further, participants had to be able to walk independently with or without a walking aid and able to speak and understand Norwegian. Exclusion of potential participants were done if they had known medical contraindications for exercising, such as severe lung diseases or progressive neurological disorders.

### Intervention

The intervention was a group-based resistance and balance circuit programme instructed by an experienced physiotherapist. The design of the programme was informed by recommendations for exercising for people with osteoporosis and vertebral fracture [13] and from exercise recommendations for older people [27]. The intervention focused on weight-bearing exercises to improve muscle strength and balance as well as strengthening exercises for the back extensors and upper arm muscles.

Each exercise session lasted 1 h and consisted of two rounds of eight different strength and balance exercises performed in a circuit, with a short warm-up before the circuit and closing with flexibility and stretching. The group had up to eight participants, and safety considerations were a priority both when designing the programme and during each session. The experienced physiotherapist was responsible for adjustments and progression of the exercises for each participant throughout the exercise period. The exercise goal was moderate intensity, corresponding to 8–12 repetitions for each exercise and a perceived level of exertion of 13 to 14 on the Borg Rating of Perceived Exertion scale [28]. The intervention is described in more detail elsewhere [24, 25] and in Supplementary File 2.

The participants allocated to the control group were instructed to live life as usual, which meant continuing with their usual everyday life and level of physical activity during the study period. Participants in the control group were offered to take part in the exercise programme after their last follow-up assessment at 6 months post-randomisation.

### Outcome measures

Trained physiotherapists, blinded to group allocation, assessed the outcome measures at baseline, at the intervention end (3 months) and at the 3-month follow-up (6 months). Background information such as age, education, body mass index, smoking status, medication, comorbidities, living alone or not, afraid of falling or not, injuries caused by falls, taking analgesics (yes/no), physical activity and pain level in the previous week (score from 0 to 10 on the Numeric Pain Rating Scale [NPRS]) was collected at baseline to describe the population. Information about walking and time spent sitting (measured by the International Physical Activity Questionnaire Short Form [IPAQ-SF]), afraid of falling (yes/no) and pain level in the previous week (measured by NPRS) was collected at the intervention end and at the 3-month follow-up.

### Primary outcome

The primary outcome of the original trial was physical fitness measured by 10-m habitual walking speed. Participants were instructed to walk 10-m from a static start at a comfortable pace (self-selected speed) [29]. This was repeated three times, and the average speed in metres per second was calculated.

### Secondary outcomes

Secondary outcomes were measures of physical fitness (balance, muscle strength and endurance), HRQoL and the Falls Efficacy Scale International (FES-I).

#### Physical fitness

The Four Square Step Test (FSST) [30] and functional reach (FR) [31] were used to assess balance. FSST is a dynamic balance test [30] and is shown to be valid and reliable when used to test community-dwelling elderly adults. A cut-off score of 15 s discriminates between multiple fallers (over 15 s) and non-multiple fallers, with a sensitivity of 85% and a specificity of 88–100% [30]. FR is a reliable and valid measure of balance [31] that measures the capacity to reach forward in an anticipatory postural adjustment task [32]. Grip strength was measured with a hydraulic handheld dynamometer [33]. Handgrip strength is a simple and reliable test for the assessment of muscle status in older adults [33–35]. Individual tests from the validated Senior Fitness Test were also applied: lower extremity leg strength (30-s sit to stand [30STS]), mobility (2.45-m up and go), upper arm strength (number of arm curls in 30 s with a 2.3-kg [5-lb] weight) and functional endurance (6-min walk test [6MWT]). The Senior Fitness Test is a valid and reliable test for physical fitness in older people and consists of several tests that assess underlying physical components associated with mobility [36].

#### HRQoL

The 36-item Short Form Health Survey (SF-36) [37] is a generic instrument for measuring HRQoL, found to be valid and reliable in the general older population [38]. It is divided into physical and mental components based on eight different subscales: physical functioning, role physical, bodily pain, general health, vitality, social function, role emotional and mental health. The score ranges from 0 to 100, with a higher score indicating better health. Further, the disease-specific Quality of Life Questionnaire of the European Foundation for Osteoporosis (QUALEFFO-41) [6] was also applied, QUALEFFO-41 has five subscales and a total score: pain, physical function, social function, general health perception, mental function and total score. The score ranges from 0 to 100, with a higher score indicating better HRQoL.

#### Other outcome measures

Fear of falling was measured by the validated Norwegian version of the FES-I [39], which measures fear of falling in 16 daily activities.

### Sample size

Sample size was calculated based on a substantial meaningful change of 0.1 m/s in 10-m habitual walking speed, with an expected SD of 0.2 m/s [40] as described in the main outcome paper [24]. To obtain 80% statistical power with a 5% significance level, 128 participants (64 per group) were required.

### Statistical analyses

All analyses were conducted using SPSS 25.0 (SPSS Corporation, Armonk, NY, USA) and Stata version 15 (StataCorp LLC, College Station, TX, USA). The data were analysed according to the intention-to-treat (ITT) principle for participants who completed the assessments. Differences between groups were assessed using linear mixed models for repeated measurements using a subject-specific random intercept and maximum likelihood estimation with the respective outcome measurement at baseline. Group, time (i.e., post-intervention and 3-month follow-up) and the interaction between group and time were fixed effects. Mixed models are considered a robust method for missing data in ITT analysis of pre–post studies [41]. The underlying structure of the model estimates the outcome at each visit, assuming that the missing data have the same correlation structure as observed data [42]. *P*-values ≤0.05 were considered statistically significant, and all tests were two sided.

## Results

### Participants

**Fig. 1:**
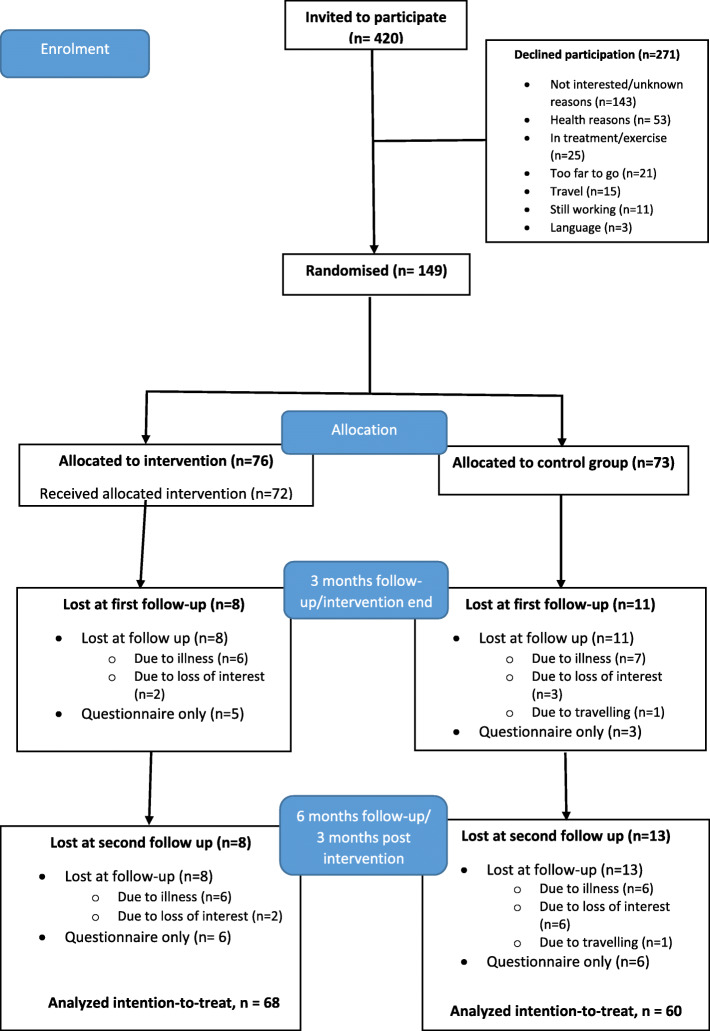
Flow of the participants throughout the study period

In total 149 participants were recruited to the original study [24], with 76 allocated to the intervention group and 73 to the control group. Eight participants in the intervention group and 11 in the control group were lost at the first (3-month) follow-up. At the second follow-up, eight participants in the intervention group and 13 in the control group were lost. Further detail on the flow of the participants is shown in Fig. 1.

At baseline, the mean age of the participants was 74.2 years, and the mean for education was 13.1 years at school. 74% of the included women answered “yes” to fear of falling, and 43.9% had experienced a fall within the last year. At 6 months (3 months post-intervention), the median walking metabolic equivalent of task (MET) measured by IPAQ-SF was 809 for the intervention group and 990 for the control group (Table 1). Time spent sitting per day was 300 min for the intervention group and 340 min for the control group. For the intervention group, the overall adherence to the exercise programme in the original study [24] was 82.6%. No serious adverse events related to the intervention were reported, but one participant withdrew from the intervention before 12 weeks due to sciatic pain (at 3 weeks), and another participant withdrew due to a flare-up of rheumatic disease (at 2 weeks). More details on adverse events not related to the intervention are reported elsewhere [24].

**Table 1:** Descriptive statistics of the sample at baseline, 3 months and 6 months^a^

	Total (*N* = 149)	Intervention’ (*N* = 76)	Control (*N* = 73)	Intervention 3 months	Control 3 months	Intervention 6 months	Control 6 months
**Characteristics**							
Age, years, mean (SD)	74.2 (5.8)	74.7 (6.1)	73.7 (5.6)				
BMI, kg/m^2^, mean (SD)	23.2 (3.7)	23.2 (3.4)	23.2(4.1)				
Smoking (Yes/No %)	10.7/89.3	14.5/81.5	6.8/93.2				
Education, mean (SD)	13.1 (3.4)	12.8 (3.2)	13.5 (3.6)				
Walking aids (Yes/No %)	19.5/80.5	18.4/81.6	20.5/79.5				
Living alone (Yes/No %)	45.1/54.9	47.3/52.7	42.9/57.1				
Comorbidity (Yes/No %)	40.5/59.5	38.7/61.3	42.5/57.5				
Painkillers (Yes/No %)	41.8/58.2	40.0/60.0	43.7/56.3				
Painkillers on prescription (Yes/No %)	58.6/41.4	53.3/46.7	64.3/35.7				
Fear of falling (Yes/No %)	74.0/26.0	75.3/24.7	72.6/27.4				
Falls last year (Yes/No %)	43.9/56.1	44.7/55.3	43.1/56.9				
IPAQ, % high	32.1	29.3	35.0				
% moderate	37.0	46.3	27.5				
% low	24.4	37.5	30.9				
IPAQ, walking MET, median	743	792	693	693	1039	809	990
225% percentile	264	297	236	264	297	380	371
50% percentile	743	792	693	693	1039	809	990
75% percentile	1386	1386	1386	1485	1518	2079	1733
IPAQ Sitting per day, minutes, mean (SD)	320 (141)	320 (149)	320 (133)	307 (131)	323 (134)	300 (129)	340 (137)
NPRS pain, mean (SD)	3.4 (2.5)	3.2 (2.2)	3.5 (2.7)	3.0 (2.1)	3.5 (2.8)	3.1 (2.2)	3.3 (2.9)
FES-1, mean (SD)	24.3 (6.7)	24.7 (6.6)	23.8 (6.8)	23.0 (5.2)	23.2 (6.7)	23.8 (6.0)	23.5 (7.6)
**Physical function, mean (SD)**							
10 m Walking speed, m/s	1.21 (0.30)	1.20 (0.29)	1.22 (0.30)	1.33 (0.26)	1.30 (0.30)	1.32 (0.28)	1.31 (0.30)
FR, cm	34.1 (6.4)	33.9 (6.2)	34.3 (6.6)	34.7 (6.6)	35.6 (8.0)	36.3 (6.4)	35.5 (8.1)
FSST, sec	9.61 (3.1)	9.80 (3.2)	9.40 (3.05)	8.93 (2.26)	9.42 (3.29)	8.77 (1.78)	9.13 (3.19)
Grip strength, right, kg	22.0 (5.1)	21.6 (4.7)	22.3 (5.4)	21.2 (4.9)	21.6 (4.5	21.1 (5.1)	21.2 (4.6)
Arm curls	15.2 (3.8)	15.1 (3.9)	15.3 (3.8)	18.2 (3.5)	17.2 (3.3)	17.8 (3.4)	16.5 (4.0)
30STS	12.6 (3.9)	12.8 (3.6)	12.5 (4.2)	14.6 (4.0)	13.0 (3.7)	14.4 (3.8)	13.3 (4.2)
2.45 m Up and Go, sec	6.51 (2.57)	6.50 (1.97)	6.52 (3.10)	6.46 (1.66)	6.82 (2.35)	6.40 (1.51)	6.75 (2.61)
6 MWD, m	471 (131)	468 (120)	473 (142)	506 (104.8)	490 (133.6)	497.6 (112.8)	506.2 (135.8)
**Health related quality of life**							
**SF-36 scores, mean (SD)**							
Physical functioning	67.6 (22.9)	66.5 (21.0)	68.7 (24.9)	70.4 (19.7)	70.2 (25.2)	69.4 (20.2)	70.7 (25.5)
Role physical	63.0 (29.0)	60.6 (27.8)	65.6 (30.1)	67.0 (28.2)	67.2 (31.1)	65.2 (25.9)	67.3 (28.4)
Body pain	58.8 (23.7)	57.6 (22.7)	60.0 (24.9)	62.9 (23.6)	64.8 (25.2)	59.2 (23.0)	60.6 (28.2)
General health	63.7 (23.3)	63.9 (22.3)	63.4 (24.4)	63.7 (20.3)	64.9 (23.0)	64.0 (20.2)	64.6 (24.7)
Vitality	53.9 (16.6)	53.2 (14.7)	54.5 (18.5)	54.3 (17.4)	57.0 (18.5)	54.1 (14.4)	53.7 (18.3)
Social function	84.1 (20.5)	85.2 (19.3)	82.9 (21.8)	86.0 (19.6)	84.9 (23.0)	83.7 (20.4)	85.2 (23.3)
Role emotional	63.1 (20.6)	63.7 (20.3)	62.6 (20.9)	67.2 (13.9)	65.4 (18.5)	67.9 (16.4)	65.3 (20.2)
Mental health	71.6 (13.1)	72.6 (10.4)	70.6 (15.5)	73.8 (9.6)	71.7 (13.9)	70.9 (11.7)	69.7 (14.8)
Physical component score	43.0 (10.0)	42.3 (9.2)	43.7 (10.8)	44.0 (9.3)	45.3 (10.6)	43.5 (9.5)	44.5 (11.4)
Mental component score	49.7 (6.6)	50.4 (5.5)	48.9 (7.5)	50.5 (5.3)	49.6 (6.7)	49.8 (5.6)	49.0 (6.6)
**QUALEFFO-41, mean (SD)**							
Pain	35.3 (25.2)	34.7 (25.0)	35.8 (25.4)	29.3 (25.9)	28.9 (24.8)	33.4 (24.0)	33.7 (26.5)
Physical function	17.2 (13.2)	17.3 (10.9)	17.2 (15.3)	14.9 (9.7)	15.5 (13.9)	16.4 (11.3)	16.7 (16.5)
Social Function	25.8 (21.1)	28.2 (20.4)	23.4 (21.7)	24.1 (17.7)	20.6 (19.1)	27.9 (21.1)	23.3 (21.7)
General Health Perceptions	44.8 (22.5)	46.6 (22.0)	42.9 (23.1)	44.0 (18.9)	44.5 (26.9)	45.2 (21.6)	42.2 (25.9)
Mental Function	34.3 (12.9)	34.0 (11.9)	34.5 (14.0)	32.9 (10.8)	33.7 (14.1)	34.3 (11.3)	35.4 (16.5)
Total score QUALEFFO	26.7 (13.1)	27.1 (11.1)	26.3 (15.0)	24.4 (10.5)	24.4 (6.7)	26.4 (11.8)	26.0 (16.6)

*n* Number of individuals, *SD* Standard deviation, *NPRS* Numeric Pain Rating Scale, *IPAQ* International Physical Activity Questionnaire (Short Form), *MET* Metabolic equivalent of task, *FES-I* Falls Efficacy Scale International, *FR* Functional reach, *FSST* Four Square Step Test, *30STS* 30-s sit to stand, *6MWD* 6-min walking distance, *SF-36* 36-item Short Form Health Survey. Comorbidity = four or more self-reported diseases

^a^There was no statistically significant difference between the intervention group and the control group on any of the descriptive variables or outcome variables at baseline

### Repeated measurements

**Fig. 2:**
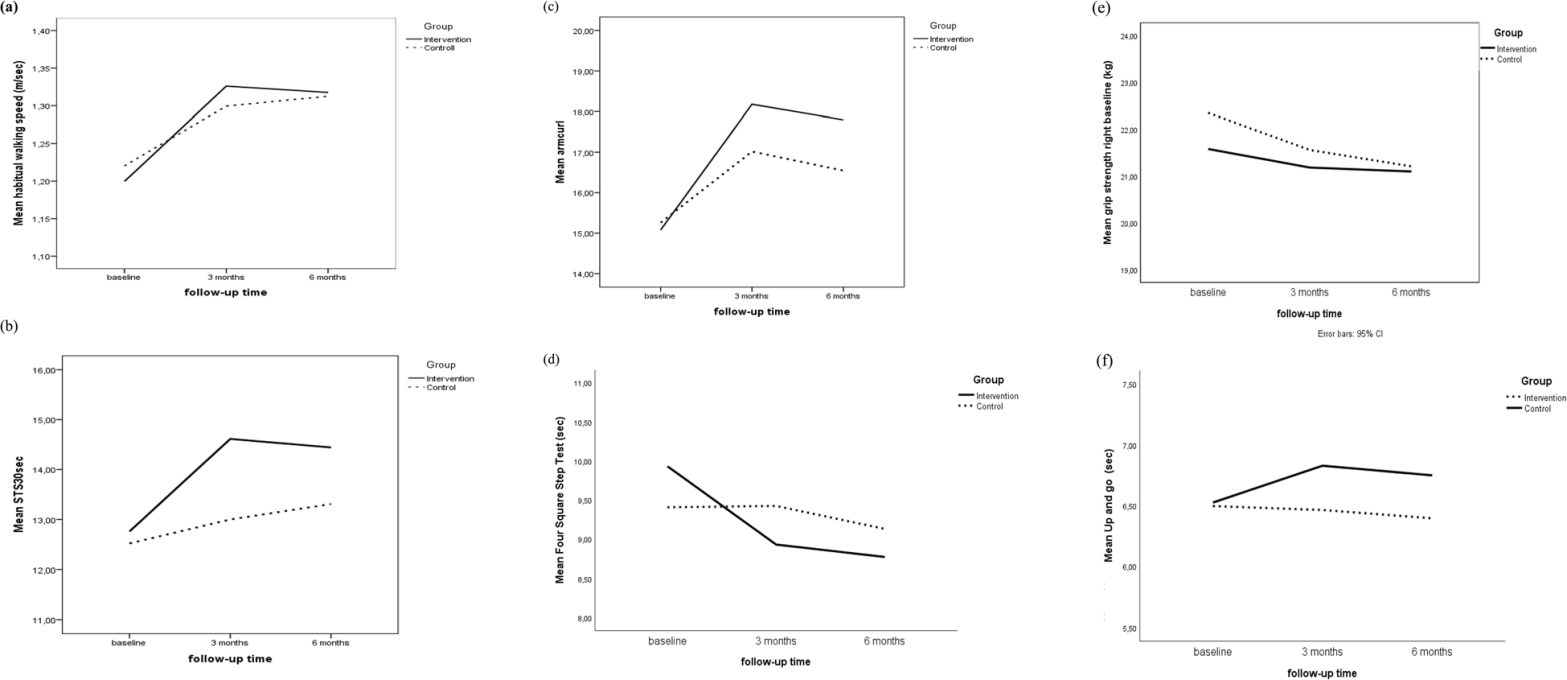
Line plots of some of the physical fitness measures at baseline, 3 months and 6 months for (**a**) walking speed, (**b**) 30STS, (**c**) arm curl, (**d**) FSST, (**e**) grip strength and (**f**) 2.45-m up and go

There was no statistically significant difference between the intervention and the control group at the 3-month follow-up for the primary outcome (Table 2) of habitual walking speed (0.03 m/s, 95%CI − 0.02 to 0.08, *p* = 0.271). However, for the secondary outcome of physical fitness, there were statistically significant differences in favour of the intervention group for balance using FSST (− 0.68 s, 95%CI − 1.24 to − 0.11, *p* = 0.019), arm curl (1.3, 95%CI 0.25 to 2.29, *p* = 0.015), leg strength using 30STS (1.56, 95%CI 0.68 to 2.44, *p* = 0.001) and mobility using 2.45-m up and go (− 0.38 s, 95%CI − 0.74 to − 0.02, *p* = 0.039). There was also a statistically significant difference between the groups regarding fear of falling in favour of the intervention group (− 1.7, 95%CI − 2.97 to − 0.38, *p* = 0.011). Regarding the HRQoL instruments, there were no statistically significant difference between the groups across all the subscales for either the generic or the disease-specific instrument (Fig. 2).

**Table 2:** Differences between groups at 3 months post-intervention, adjusted for baseline values. Mean difference refers to intervention-control

Outcomes	Mean difference	95%CI	*P* value
Habitual walking speed	0.03	−0.02 to 0.08	0.271
FR	1.54	−0.68 to 3.77	0.173
FSST	−0.68	−1.24 to − 0.11	**0.019**
Grip strength right	0.07	−0.99 to 1.13	0.895
Arm curl	1.27	0.25 to 2.29	**0.015**
30STS	1.56	0.68 to 2.44	**0.001**
2.45-m up and go	−0.38	−0.74 to − 0.02	**0.039**
6MWD	−1.06	−19.66 to 17.54	0.911
**HRQoL**			
*SF-36*			
Physical functioning	1.73	−3.21 to 6.66	0.490
Role physical	2.69	−3.71 to 9.10	0.408
Bodily pain	0.88	−3.88 to 5.64	0.717
General health	−0.00	−4.73 to 4.73	0.999
Vitality	1.84	−2.05 to 5.73	0.353
Social functioning	−0.86	−5.76 to 4.05	0.732
Role emotional	1.90	−3.10 to 6.91	0.042
Mental health	0.01	−3.43 to 3.44	0.997
Physical component score	0.82	−2.64 to 1.00	0.374
Mental component score	−0.06	−1.78 to 1.66	0.944
*QUALEFFO-41*			
Pain	0.66	−6.06 to 4.74	0.810
Physical function	−1.12	−1.56 to 3.80	0.409
Social function	−0.39	−5.17 to 4.38	0.871
General health perceptions	0.32	−5.08 to 5.65	0.906
Mental function	−1.42	−4.26 to 1.41	0.323
Total QUALEFFO-41 score	0.96	−3.36 to 1.44	0.431
NPRS	0.11	−0.46 to 0.68	0.709
FES-I	−1.68	−2.97 to −0.38	**0.011**

*FR* Functional reach, *FSST* Four Square Step Test, *30STS* 30-s sit to stand, *6MWD* 6-min walking distance, *HRQoL* Health-related quality of life, *SF-36* 36-item Short Form Health Survey, *QUALEFFO-41* Quality of Life Questionnaire of the European Foundation for Osteoporosis, *NPRS* Numeric Pain Rating Scale, *FES-I* Falls Efficacy Scale International. Mean difference refers to intervention minus control

## Discussion

We have established that in older women with osteoporosis and vertebral fracture, the effects of a 3-month exercise programme on upper and lower limb muscle strength, balance, mobility and fear of falling are maintained, at least in part, at 3 months post-intervention. However, there were no between-group differences evident in habitual walking speed or HRQoL. Results from the present study are consistent with other works suggesting that exercise may have a sustainable effect on balance, mobility [15] and fear of falling [17] in older women with osteoporotic vertebral fractures.

In contrast to our hypothesis, the exercise intervention did not have an effect on the primary outcome – habitual walking speed – either at the end of the intervention [24] or at the 3-month follow-up. Walking speed is considered a robust tool for measuring physical capacity, and has extensive predictive capabilities, e.g. on outcomes like functional dependence, mobility and falls [29]. At baseline, the participants in the present study had an average habitual walking speed of 1.21 m/s. Compared to different cut-off values for walking speed among older people [29], the walking speeds observed in our study correspond to a functional level in which individuals can manage their daily tasks well, are independent in self-care and have ambulation in the community. Therefore, we may have observed a ceiling effect for habitual walking speed [43], and any further increase may have been difficult to achieve. In contrast, Bergland et al. [15] reported improved maximum walking speed after a 3-month exercise intervention, which persisted even after cessation of exercise at the 12-month follow-up in older women with osteoporosis and vertebral fracture. Habitual walking speed, also referred to as normal walking speed, provides information about an individual’s mobility, whereas measuring maximum walking speed is associated with muscle power in the lower extremities [44]. Therefore, maximum walking speed might have been a more sensitive outcome measure, which is supported by our findings showing a significant increase in lower limb and upper limb muscle strength in the short term.

The improved muscle strength among the participants in the intervention group at the 3-month post-intervention follow-up is interesting, as we know that muscle strength naturally declines with age [20]. Our findings are in line with those of a recent randomised controlled trial of home exercises in older women with vertebral fracture, which reported improved functional leg muscle strength after 12 months [18]. Maintenance of muscle strength may prevent loss of functional dependence [27]. A systematic review reported that exercise may prevent falls and fall-related fractures and reduce risk factors for falls in individuals with low bone mineral density [45]. However, individuals with vertebral fractures may have pain or hyperkyphosis that modifies the effect of exercise on fall or fracture risk. The effect of exercise on mobility as measured by the 2.45-m up and go is promising, but the magnitude of the effect was small (between-group difference of 0.38 s) and likely not clinically meaningful [46]. Maintaining physical activity and physical function is important for people with osteoporosis [47] and may prevent a cycle of physical impairment after vertebral fracture [1, 8, 48, 49].

In contrast to previous studies [15, 16, 50], we found no effect of exercise on HRQoL (using either the generic or the disease-specific instrument) at the 3-month post-intervention follow-up. Evstigneeva et al. [50] and Bergland et al. [15] both reported statistically significant improvements in QUALEFFO-41 score in favour of the exercise intervention group after 12 months of exercise. However, compared to other studies in the same population, the participants in our study had higher scores for both the generic SF-36 and the disease-specific QUALEFFO-41 [8, 15, 50]; thus, we may have experienced ceiling effects. Several studies show that osteoporosis has a negative effect on HRQoL [7], and within the population with osteoporosis, people with vertebral fracture report moderately lower physical health status compared to osteoporotic people without vertebral fracture [7]. Therefore, there is merit in identifying how to improve HRQoL among those with osteoporosis and low self-reported HRQoL.

### Strengths and limitations

One of the strengths of our study was its design as a single-blinded randomised controlled trial. We published a study protocol a priori which elaborated on the background, rationale for the study, assessment of outcomes and how the intervention would be carried out [25]. An available study protocol can reduce publication bias and improve reproducibility [26]. Furthermore, the intervention applied was informed by exercise recommendations for people with osteoporosis or osteoporotic vertebral fractures [13] and described according to the Consensus on Exercise Reporting Template (CERT -statement [51].

Some limitations of the study should be mentioned. Information about exercise habits or frequency in the intervention and control groups could have been explored in more detail. The follow-up time of the present study was short, which restricts our ability to make inferences regarding maintenance effects of exercise beyond 3 months post-intervention. Number, location and severity of vertebral fractures are associated with pain, disability and HRQoL [8, 52] and could add valuable information for interpretation and generalisability of the results. Unfortunately, this information was not available to us, neither was information regarding the participants anti-osteoporotic drug prescriptions.

Finally, our sample was a relatively healthy group of individuals with vertebral fractures living at home. Our findings may not be generalisable in individuals living in assisted settings, individuals who are more frail or individuals who have cognitive impairment, as the curve of decline in physical function may be steeper and affected by age and number of vertebral fractures [21]. Physical and cognitive impairments may have made it difficult to participate or adhere to the intervention.

## Conclusions

In conclusion, the positive effects of a resistance and balance exercise programme on physical fitness (e.g., muscle strength, balance and mobility) and fear of falling persisted to some extent 3 months after cessation of exercise in older women with osteoporosis and vertebral fracture. There was no effect on walking speed or HRQoL at follow-up.

## Supplementary information

**Additional file 1.** CONSORT 2010 checklist of information to include when reporting a randomised trial.

**Additional file 2.** Detailed description of the intervention following the CERT-guidelines.

## Data Availability

The datasets generated and/or analysed during the study are only available to the participating researchers due to data protection laws. Subsets or aggregation of these data will not include information that could compromise research participants’ privacy. Consent can be made available from the corresponding author on reasonable request.

## Abbreviations

30STS: 30-s sit to stand
6MWT: 6-min walk test
DXA: Dual-energy x-ray absorptiometry technology
FES-I: Falls Efficacy Scale International
FR: Functional reach
FSST: Four Square Step Test
HRQoL: Health-related quality of life
IPAQ-SF: International Physical Activity Questionnaire Short Form
ITT: Intention to treat
MET: Metabolic equivalent of task
NPRS: Numeric Pain Rating Scale
QUALEFFO-41: Quality of Life Questionnaire of the European Foundation for Osteoporosis
SF-36: 36-item Short Form Health Survey
