# Supplementary material for: Physical fitness in older women with osteoporosis and vertebral fracture after a resistance and balance exercise programme: 3-month post-intervention follow-up of a randomised controlled trial
Source: BMC Musculoskelet Disord. 2020 Jul 18;21:471. doi: 10.1186/s12891-020-03495-9 (PMC7368978; doi:10.1186/s12891-020-03495-9)
Supplement: Supplementary file 2 — Additional file 2. Detailed description of the intervention following the CERT-guidelines. [file 12891_2020_3495_MOESM2_ESM.docx]

**Additional file 1:** CERT- guidelines

Detailed description of the exercise intervention applied in the study “ A resistance and balance exercise programme on physical fitness, health related quality of life and fear of falling in older women with osteoporosis and vertebral fracture: a randomized controlled trial”, reporting follows the CERT guidelines. *Slade SC, Dionne CE, Underwood M, et al, Consensus on Exercise Reporting Template (CERT): Explanation and Elaboration Statement*

*British Journal of Sports Medicine 2016;****50:****1428-1437.*

| Section/topic | Item # | Checklist item | Location | |
| --- | --- | --- | --- | --- |
|  |  |  | Original paper | Protocol paper |
| What: Materials | 1 | Reebok® Steps, rubberbands with low, medium and high resistance, Airex® balance pad, chairs, handheld weightmanuals 1-5 kilos, training mats and weightbelts carrying up to 6 kilos were used as equipment for the exercises. |  | Page 4 |
| Who: Provider | 2 | Four physiotherapists delivered the intervention, with clinical experience ranging from 4 to 27 years. All physiotherapists went through detailed training and instruction regarding the specific exercises of the intervention and participated at least two intervention-sessions together with the person responsible for the training (project leader). | Page 4 | Page 4 |
| How: Delivery | 3 | The participants exercised in groups of up to 8 women. The groups were running continuously, with participants in different stages of their 12 weeks period. All participants were subscribed 12 weeks of exercise, twice a week (in total 24 sessions). When there were more than 2 women starting at the same time, a second physiotherapist co-instructed the session to ensure individual tailoring and instruction for those who were new to the intervention. | Pages 4 -5 | Page 4 |
|  | 4 | All exercise sessions were supervised and instructed by a physiotherapist. The physiotherapist instructed the warm-up session. During the main session of the resistance and balance training, the physiotherapist provided face-to face feedback, guidance and motivation, observed exercise performance, corrected exercise technique and modified exercises. | Pages 4-5 |  |
|  | 5 | Adherence was described as the percentage of exercise sessions completed, divided by the number of exercise session subscribed (24). Adherence to exercise were recorded in an attendance scheme at every session by the physiotherapist who instructed the group. | Page 8 | Page 6 |
|  | 6 | All participants were encouraged and motivated verbally during exercise sessions by the physiotherapist. If the participant didn’t attend for unknown reasons, phone calls were made to motivate for returning to exercise. |  |  |
|  | 7a | For a gentle start-up of the exercise programme, each participant was instructed to only do one round of the exercise circuit for the first two weeks in order to minimize muscle soreness and other minor adverse events. The exercise goal for the first two weeks was to reach a light to moderate training level, (11-12 out of 20 on Borg rating scale of perceived exertion, which correspond to up to 60 % maximum heart rate). After two weeks the exercise goal was adjusted to moderate intensity level, corresponding to 8-12 RM for each exercise, and a perceived level of exertion of 13-14 on the Borg rating of Perceived Exertion scale ^1^. The participants were encouraged to work until volitional fatigue so that they perceived it as somewhat hard. | Page 5 | Page 4 |
|  | 7b | The exercise intensity was adjusted based on the observation of the physiotherapist and the participants perceived intensity level. Progression was made by increasing weights (manuals or load in the weight belts), increasing frequency, advancing to higher resistance on rubber band, higher height on steps, smaller base of support in balance, or more challenging positions (from push-up against the wall to push up on a lower bench). Progression throughout the exercise period (12 weeks) was emphasized, and the physiotherapist played an important part in maintaining progression through observation and encouraging of the participants. | Page 5 |  |
|  | 8 | \| **Exercise** \| **Therapeutic goal** \| **Level** \| **Description** \| **Progression or modify** \| \| --- \| --- \| --- \| --- \| --- \| \| Squats \| Leg strength \| 1 \| Raise from a chair (sit to stand) \| Tempo,  weightbelt \| \|  \|  \| 2 \| Deep squats (lower than 90 degrees kneeflexion) \| Weightbelt,  Degrees of kneeflexion \| \|  \|  \| 3 \| Lounges \| Weightbelt \| \| Step up \| Leg strength, dynamic balance \| 1 \| Go up and down from a step, change legs \| Height of steps, tempo,  Weightbelt. support \| \|  \|  \| 2 \| Step up and one leg stance, go down \| Tempo, height of steps \| \| Sideways step up \| Hip stability, dynamic balance, leg strength \| 1 \| Step up sideways on a step \| Height of the step, tempo, weightbelt \| \| Upright row \| Posture, upper back strengthening \| 1 \| Row exercise with elastic bands \| Three different resistance levels on the elastic bands \| \| Balance pad \| Balance, static and dynamic \| 1 \| Standing on balance pad, semitandem \| Open/closed eyes, turning head from one side to the other \| \|  \|  \| 2 \| Standing on balance pad, tandem \|  \| \|  \|  \| 3 \| One leg standing \|  \| \| Chest press \| Chest and arm strengthening \| 1 \| Chest press against the wall \|  \| \|  \|  \| 2 \| Chest press against a bench \|  \| \|  \|  \| 3 \| Chest press resting on the knees on the floor \|  \| \| Hip raise \| Lower back strengthening \| 1 \| Lying on back with knees bent, raise hip \|  \| \|  \|  \| 2 \| One leg hip raise \|  \| \| Diagonal lift \|  \| 1 \| Standing on all four, lift left arm and right leg, alternate \| Choose either hip raise or diagonal lift \| \| Biceps curl \| Upper arm strengthening \| 1 \| Seated armcurls with dumbbells \| Increase load of dumbbells  (1, 2, 3, 4 or 5 kg) \| |  | Table 1, page 5 |
|  | 9 | There were no home program components belonging to the intervention. |  |  |
|  | 10 | There were no non-exercise components in the intervention |  |  |
|  | 11 | Adverse events were recorded by the physiotherapist after each session. There were no serious adverse events related to the intervention, but some experienced short-term muscle soreness and joint pain. One participant had a fall at the facilities before the exercise session started, but with no consequences regarding pain or injuries. Some participants in the intervention group experienced adverse events unrelated to the intervention; humerus fracture (n=1), concussion after a fall (n=1), pneumonia (n=2), pelvic fracture (n=1), falls (n= 7), flare up of rheumatic pain (n=1). Adverse events in the control group were not recorded. | Pages 11-12 | Page 4 |
|  | 12 | The intervention was delivered in a clinical setting at two different out-patient clinics in rooms suitable for exercising. | Page 4 | Page 3 |
|  | 13 | The exercise session started with 10 min warm- up focusing on flexibility, balance and dynamic exercises of muscle groups in the legs and upper body instructed by the physiotherapist. The warm up was follow by two rounds of resistance and balance exercises, organized as a stationary circuit setup. The participants worked for 1.5 minutes at each station, with a short break of 30 s to rest and move to the next station. After one round of eight exercises, there was a break of 2 -3 minutes to drink water and rest, before a second round at the eight different stations. The participants were instructed to work through the whole period (1.5 min) and keep the highest intensity they were capable of, given their individual capacity. For the balance exercise, instructions were given to challenge their balance the best they could without jeopardizing safety. The session ended with 10 minutes cool-down and stretching instructed by the physiotherapist. The whole session lasted 1 hour. | Page 4 and 5 | Page 4 |
|  | 14a | We developed an exercise program based on exercise recommendations for older people with osteoporosis and vertebral fracture ^2^. The exercises were evaluated according to recommendations and safety cautions, and exercises with flexion or rotation of the spine were excluded. It was also emphasized that all exercise stations had support objects, for prevention of falls. The program includes weightbearing strengthening exercises for the lower limbs which also have components of dynamic balance (3 different exercises), exercises for strengthening of upper and lower back (2), strengthening of upper limb (2) and a balance exercise. (See more details in the table in item 8) | Page 4 and 5 | Page 3 |
|  | 14b | The exercises were individualized and adapted if the participants had comorbidities that made it difficult to do the exercise the way it was subscribed. The physiotherapist encouraged progression when the level was under 13-14 on Borg rating scale of perceived exertion for the participant. |  |  |
|  | 15 | The starting level of the participant was partly decided through information from the participant on their exercise habits the last 6 months and a general rule that all new participants only should carry out one round of the circuit exercise (eight exercises) the two first weeks. All exercises could be performed at different levels of difficulty, basic, intermediate and advanced. The level for each participant were decided by the physiotherapist through instruction and observation of quality of the performance (correct technique) and by the perceived level of exertion the participant experienced. |  |  |
| How well: planned, actual | 16a | The intervention was delivered as intended. The physiotherapist who held the intervention went through training of the content of the intervention, as well as participating in the intervention together with the project leader twice before leading the intervention on their own. |  |  |
|  | 16b | The overall adherence in the intervention group was 82.6 % (24 sessions = 100 %), and 57 of the women (75 %) had an adherence of 80 % or more (more than 19 sessions). | Page 8 |  |

*^1^ Borg GA (1982) Psychophysical bases of perceived exertion. Medicine and science in sports and exercise 14 (5):377-381*

*^2^ Giangregorio LM, Papaioannou A, Macintyre NJ, Ashe MC, Heinonen A, Shipp K, Wark J, McGill S, Keller H, Jain R, Laprade J, Cheung AM (2014) Too Fit To Fracture: exercise recommendations for individuals with osteoporosis or osteoporotic vertebral fracture. Osteoporosis international : a journal established as result of cooperation between the European Foundation for Osteoporosis and the National Osteoporosis Foundation of the USA 25 (3):821-835. doi:10.1007/s00198-013-2523-2*
